# Tertiary Lymphoid Structures Gene Signature Predicts Prognosis and Immune Infiltration Analysis in Head and Neck Squamous Cell Carcinoma

**DOI:** 10.2174/0113892029278082240118053857

**Published:** 2024-01-29

**Authors:** Aiyan Xing, Dongxiao Lv, Changshun Wu, Kai Zhou, Tianhui Zhao, Lihua Zhao, Huaqing Wang, Hong Feng

**Affiliations:** 1 Department of Pathology, Shandong University Qilu Hospital, Jinan, Shandong, 250012, China;; 2 Cancer Center, Shandong Provincial Hospital, Cheeloo College of Medicine, Shandong University, Jinan, Shandong, 250021, China;; 3 Cancer Center, Shandong Provincial Hospital Affiliated to Shandong First Medical University, Jinan, Shandong, 250021, China;; 4 Department of Surgery, Shandong Provincial Hospital, Cheeloo College of Medicine, Shandong University, Jinan, Shandong, 250021, China;; 5 Department of Surgery, Shandong Provincial Hospital Affiliated to Shandong First Medical University, Jinan, Shandong, 250021, China;; 6 Department of Translational Medicine, Genecast Biotechnology Co., Ltd, Wuxi, Jiangsu, 214104, China;; 7 Department of Medical Oncology, Tianjin Union Medical Center, The Affiliated Hospital of Nankai University, Tianjin, 300000, China

**Keywords:** Tertiary lymphoid structure signature, head and neck squamous cell carcinoma, immune microenvironment, driver mutations, prognosis, TP53 mutation

## Abstract

**Objectives:**

This study aims to assess the prognostic implications of gene signature of the tertiary lymphoid structures (TLSs) in head and neck squamous cell carcinoma (HNSCC) and scrutinize the influence of TLS on immune infiltration.

**Methods:**

Patients with HNSCC from the Cancer Genome Atlas were categorized into high/low TLS signature groups based on the predetermined TLS signature threshold. The association of the TLS signature with the immune microenvironment, driver gene mutation status, and tumor mutational load was systematically analyzed. Validation was conducted using independent datasets (GSE41613 and GSE102349).

**Results:**

Patients with a high TLS signature score exhibited better prognosis compared to those with a low TLS signature score. The group with a high TLS signature score had significantly higher immune cell subpopulations compared to the group with a low TLS signature score. Moreover, the major immune cell subpopulations and immune circulation characteristics in the tumor immune microenvironment were positively correlated with the TLS signature. Mutational differences in driver genes were observed between the TLS signature high/low groups, primarily in the cell cycle and NRF2 signaling pathways. Patients with TP53 mutations and high TLS signature scores demonstrated a better prognosis compared to those with TP53 wild-type. In the independent cohort, the relationship between TLS signatures and patient prognosis and immune infiltration was also confirmed. Additionally, immune-related biological processes and signaling pathways were activated with elevated TLS signature.

**Conclusion:**

High TLS signature is a promising independent prognostic factor for HNSCC patients. Immunological analysis indicated a correlation between TLS and immune cell infiltration in HNSCC. These findings provide a theoretical basis for future applications of TLS signature in HNSCC prognosis and immunotherapy.

## 1 INTRODUCTION

Head and neck squamous cell carcinoma (HNSCC) is a malignant tumor that suppresses immune surveillance mechanisms, with a mortality rate ranging from 40% to 50% and a five-year survival rate of approximately 60% [1, 2]. The majority of HNSCC cases are diagnosed as locally advanced diseases [3, 4]. It has been reported that cancer immunotherapy has benefited patients with advanced cancer [5], as exemplified by Japan's approval in 2020 of near-infrared light immunotherapy for the treatment of unresectable locally advanced or recurrent HNSCC [6]. Furthermore, adjunctive combined immunotherapy with immune checkpoint inhibitors (ICI) has been demonstrated to significantly enhance clinical outcomes and prolong overall survival (OS) [5, 7, 8]. However, despite the encouraging clinical results, not all patients with HNSCC can derive benefits from immunotherapy [9]. In contrast to the success of ICI in other solid tumors, such as melanoma, the response rate in HNSCC patients is confined to a small subset (13-18%) of individuals [5, 10-13]. Therefore, the development of an effective biomarker for predicting immunotherapeutic response is of paramount importance.

Tumor-infiltrating lymphocytes (TIL), ubiquitous in various solid tumors, have emerged as crucial indicators of disease-specific survival and prognosis. Studies suggest that elevating the expression of tumor-infiltrating B cells (TIL-B) and tertiary lymphoid structures (TLS) may potentiate antitumor immunity, offering a novel avenue for T cell-based immunotherapy [14, 15]. TLS, characterized as ectopic lymphoid formations in non-lymphoid tissues, exhibits enhanced efficiency in suppressing inflammatory responses compared to secondary lymphoid organs [10, 16-19]. Furthermore, TLS serves as promising biomarkers for stratifying the risk of OS in untreated patients and as markers for effective immunotherapy [20-22]. Calderaro *et al*. reported that TLS in hepatocellular carcinoma is linked to a lower risk of early recurrence following surgical resection and suggested effective antitumor immunity *in situ* [23]. Zhou *et al*., elucidated that TLS signatures hold the potential to inform clinical decision-making and guide the treatment of cancer patients [24]. It has been shown that TIL-rich HNSCC associated with human papillomavirus has unique B- cell signatures and contributes to the prognosis of patients with HNSCC [17, 25]. Nevertheless, the genetic characterization of TLS in HNSCC remains an outstanding area for investigation.

In this study, we investigated the correlation between TLS signatures and the tumor microenvironment, driver gene mutation status, and prognostic implications in HNSCC patients using data from the Cancer Genome Atlas (TCGA) database. Additionally, we substantiated the prognostic and immune characteristics associated with the TLS signature in an independent cohort sourced from the Gene Expression Omnibus (GEO) database.

## 2 MATERIALS AND METHODS

### 2.1 Data Collection and Processing

This study is based on data from TCGA and GEO databases, which stand as prominent public repositories, offering invaluable research material in the field of genomics. Numerous high-quality studies have been published using these resources [26-29]. Gene expression profiles, single nucleotide variant (SNV) data, and clinical data were obtained for HNSCC patients (excluding HPV-associated oropharyngeal cancer) from the TCGA database (https://gdc.cancer.gov/about-data/publications/panimmune) [30]. A total of 488 cases were selected, and those without clinical follow-up data were excluded, leaving 483 cases with available DNA and RNA testing as well as clinical data (Table **1**). Fig. (**1**) presents the study flowchart. Validation datasets (GSE41613 and GSE102349) were sourced from the GEO database (https://www.ncbi.nlm.nih.gov/geo/) [8, 31]. GSE 41613 encompasses 97 samples to affirm the impact of TLS signature on the immune microenvironment in HNSCC patients. GSE102349 comprises 113 samples, encompassing 88 patients with comprehensive prognostic data, employed to validate the prognostic predictive capacity of the TLS signature. This dataset also incorporates 80 patients with stromal and intratumoral TLS data, enabling the differentiation of stromal and intratumoral TLS effects on prognosis.

To quantify the immune cell proportions in the tumor microenvironment, single-sample gene set enrichment analysis (ssGSEA) [32] was performed to evaluate 28 immune cell types. Identification of immune cell subpopulations was based on previous research [33]. The following eight axes of the immunogram score (IGS) provided descriptions of the stages of the cancer-immunity cycle: IGS1, T cell immunity; IGS2, tumor antigenicity; IGS3, priming and activation; IGS4, trafficking and infiltration; IGS5, tumor antigen identification; IGS6, inhibitor cells; IGS7, checkpoint expression; and IGS8, inhibitory molecules. These axes and their respective gene sets were used in antecedent research [34].

### 2.2 Bioinformatics Analysis

The ClusterProfiler package in the R environment was utilized to perform Gene Ontology (GO) analysis for statistical computing and graphics. Kyoto Encyclopedia of Genes and Genomes (KEGG) pathway enrichment analysis was conducted using the R package ClusterProfiler, and the false discovery rate (FDR) was controlled at a threshold of <0.1. The tumor mutational burden (TMB) value was calculated using the R package maftool.

### 2.3 Statistical Analysis

Statistical analyses were carried out using R software version 3.4.2. Data were presented as median and interquartile range (IQR). The Wilcoxon rank-sum test was employed to assess differences between the values of two groups, while the Kruskal-Wallis test was applied when examining differences among more than two groups. The log-rank test and Kaplan-Meier curves were used to compare OS and progression-free survival (PFS). Univariate and multivariate analyses were conducted using Cox proportional hazards regression, and variables with *P* < 0.1 in the univariate Cox analysis were included in the multivariate Cox analysis. A *P*-value of <0.05 was considered significant with a two-sided test.

## 3 RESULTS

### 3.1 TLS Signature and Prognosis of HNSCC Patients

**Fig. (2):**
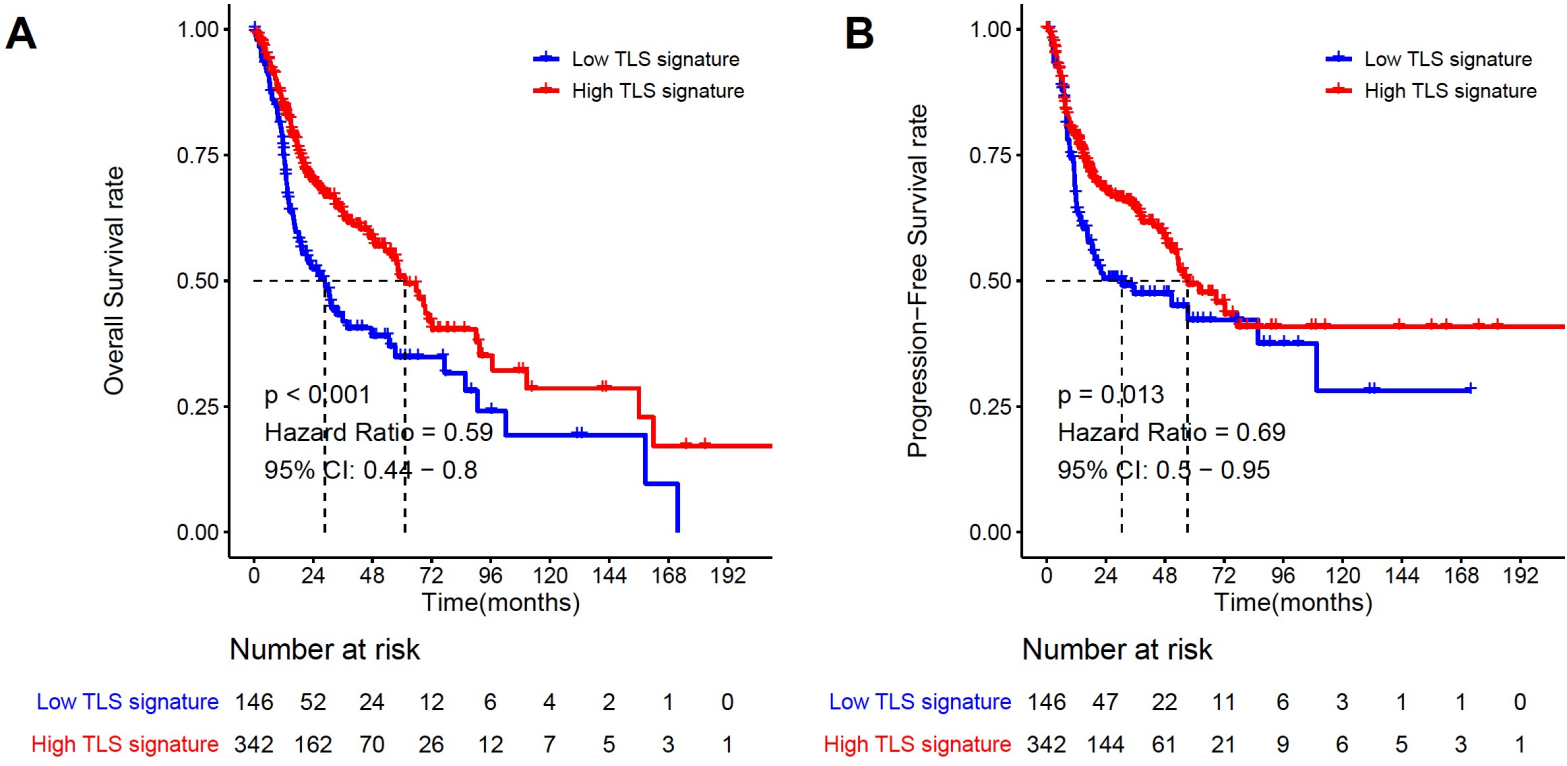
Survival analysis of HNSCC patients in both groups with high/low TLS signature. (**A**). Kaplan–Meier plots of the difference of OS between tumors with TLS signature high (n = 342) and low groups (n = 146) in HNSCC; (**B**). Kaplan–Meier plots of difference of PFS between tumors with high (n = 342) and low (n = 146) TLS signature groups in HNSCC. *P *< 0.05 was considered a significant difference.

To analyze the role of TLS signature in HNSCC, we initially evaluated the expression of nine genes in the TLS signature in both HNSCC tumor tissue and normal tissue. The expressions of CD1D (*P *< 0.01), CETP (*P *< 0.001), LAT (*P *< 0.001), and RBP5 (*P *< 0.001) in the TLS signature were significantly higher in tumors, whereas PTGDS (*P *< 0.001) exhibited the opposite trend (Fig. **S1**). Employing a threshold of less than 30% for the TLS signature, we categorized the patients into high/low TLS signature groups and plotted Kaplan-Meier curves. The results showed that the OS (*P *< 0.001, Fig. **2A**) and PFS (*P*=0.013, Fig. **2B**) of HNSCC patients with high TLS signatures were superior to those with low TLS signatures. Furthermore, both univariate and multivariate analyses were performed using the Cox proportional hazards regression. The findings revealed that the TLS signature was significantly associated with OS (*P*=0.002) and PFS (*P*=0.014), indicating that the TLS signature was an independent prognostic risk factor for patients with HNSCC (Table **2**).

### 3.2 Association of TLS Signature with the Immune Microenvironment

**Fig. (3):**
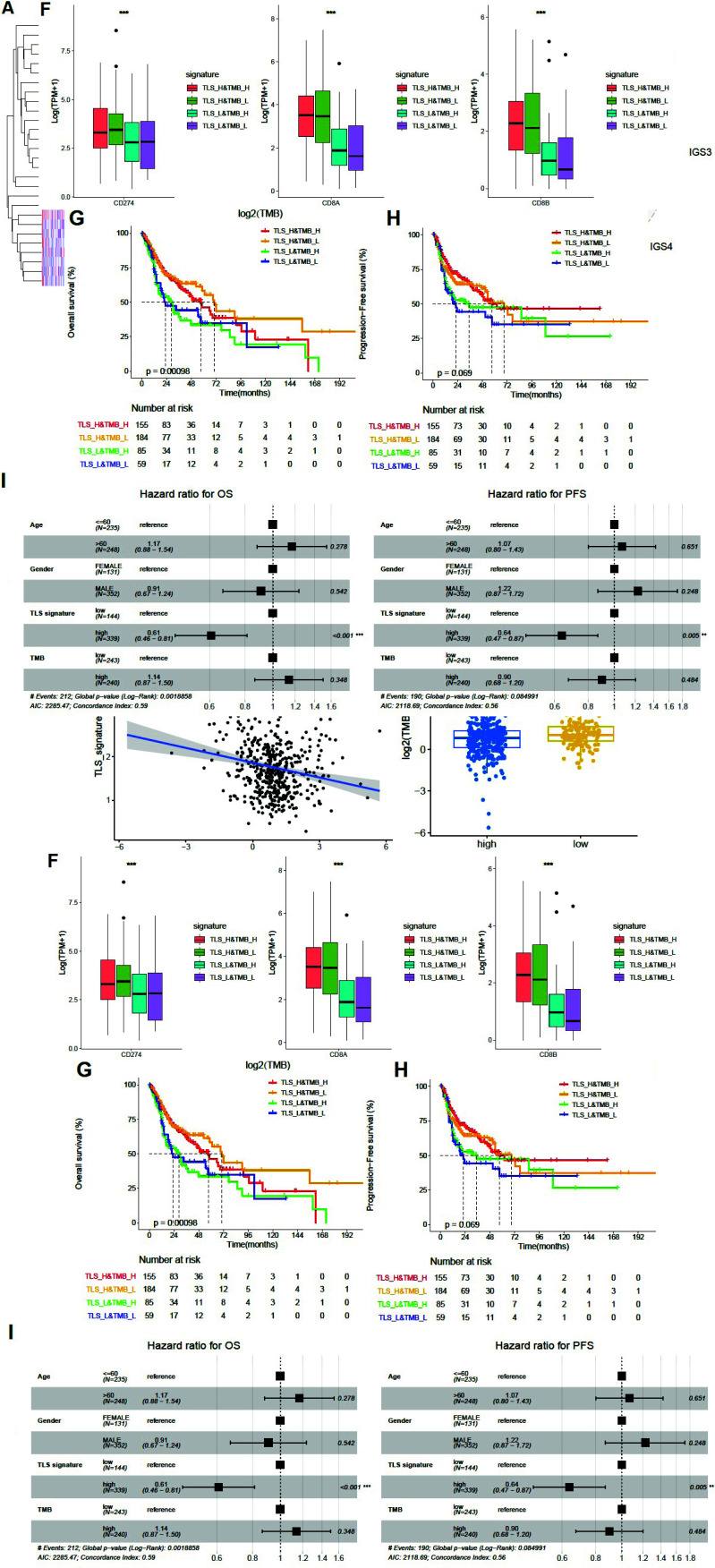
Association of TLS signature with immune microenvironment and TMB. (**A**). Differences in immune cell subsets between the groups with high TLS signature (n = 342) and low TLS signature (n = 146); (**B**). Differences in cancer immune circulation between the groups with high TLS signature (n = 342) and low TLS signature (n = 146); (**C**). Difference in checkpoint genes expression between the groups with high TLS signature (n = 342) and low TLS signature (n = 146). *P *< 0.05 was considered a significant difference, **P < 0.01, ***P < 0.001. (**D**). A relationship between TLS signature and TMB; (**E**). Differences in TMB between the groups with high TLS signature (n = 339) and low TLS signature (n = 144); (**F**). Differences in PD-L1 expression and CD8 gene expression among groups with high TLS signature and high TMB (TLS_H&TMB_H, n = 155), group with high TLS signature and low TMB (TLS_H&TMB_L, n = 184), group with low TLS signature and high TMB (TLS_L&TMB_H, n = 85), and group with low TLS signature and low TMB (TLS_L&TMB_L, n = 59); ***P < 0.001. (**G**). Differences among the four indicated groups in OS; (**H**). PFS difference among the four indicated groups. (**I**). Cox multivariate analysis of patient OS and PFS. *P *< 0.05 was considered a significant difference.

The overall differences in immune cell subpopulations between the high and low TLS signature groups are presented in Fig. (**3A**). The group with high TLS signatures exhibited significantly higher immune cell subset infiltration than the group with low TLS signatures. Moreover, most tumor-infiltrating immune cells were positively correlated with the TLS signature (Fig. **S2**). Five immune circulating features (IGS1, IGS3, IGS4, IGS6, and IGS7) were significantly higher in the group with high TLS signatures than in the group with low TLS signatures, and all of them were positively correlated with the TLS signature (*P *< 0.01, Figs. **3B** and **S3**). Furthermore, the detection of major checkpoint gene expression between the high and low TLS signature groups revealed that immunotherapy may have better therapeutic effects for patients in the group with high TLS signatures (*P *< 0.01, Figs. **3C** and **S4**).

### 3.3 Association of TLS Signature with TMB

The results of the correlation analysis indicate a negative correlation between TLS signature and TMB (*R*=-0.19, *P *< 0.001, Fig. **3D**). Patients with high TLS signatures had slightly lower TMB than those with low TLS signatures (*P*=0.0018, Fig. **3E**). HNSCC patients were categorized into four groups based on TMB (with median value as cutoff) and TLS signature levels: high TLS signature and high TMB (TLS_H&TMB_H, n=155), high TLS signature and low TMB (TLS_H&TMB_L, n=184), low TLS signature and high TMB (TLS_L&TMB_H, n=85), and low TLS signature and low TMB (TLS_L&TMB_L, n=59). There were significant differences in PD-L1 and CD8 expression among the four groups (*P *< 0.001, Fig. **3F**). Furthermore, there was a substantial difference in OS among these four groups, with patients in the TLS_H&TMB_L group having the best survival (*P*=0.00098, Fig. **3G**). However, there was no significant difference in PFS among the four groups (Fig. **3H**). Cox multivariate analysis, adjusted for TLS score, TMB group, age, and gender, demonstrated that TLS score was an independent prognostic factor for patients' OS and PFS (Fig. **3I**).

### 3.4 Association of TLS Signature with Driver Gene Mutations

**Fig. (4):**
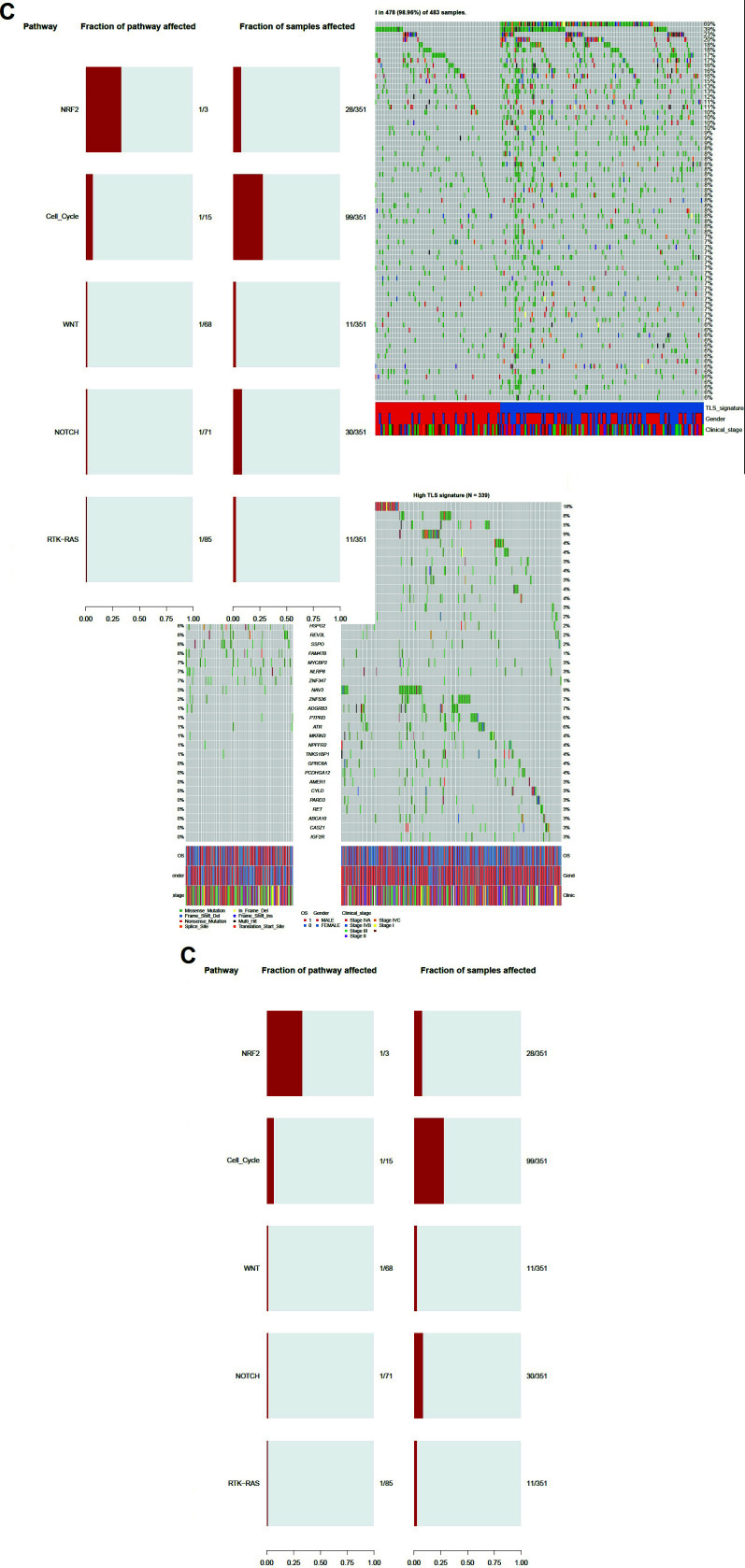
Association of TLS signature with driver gene mutations. (**A**). Panoramic display of mutations in HNSCC; (**B**). Driver genes with significantly different mutation frequencies in both TLS signature high/low groups; (**C**). Enrichment analysis of differential gene pathway.

**Fig. (5):**
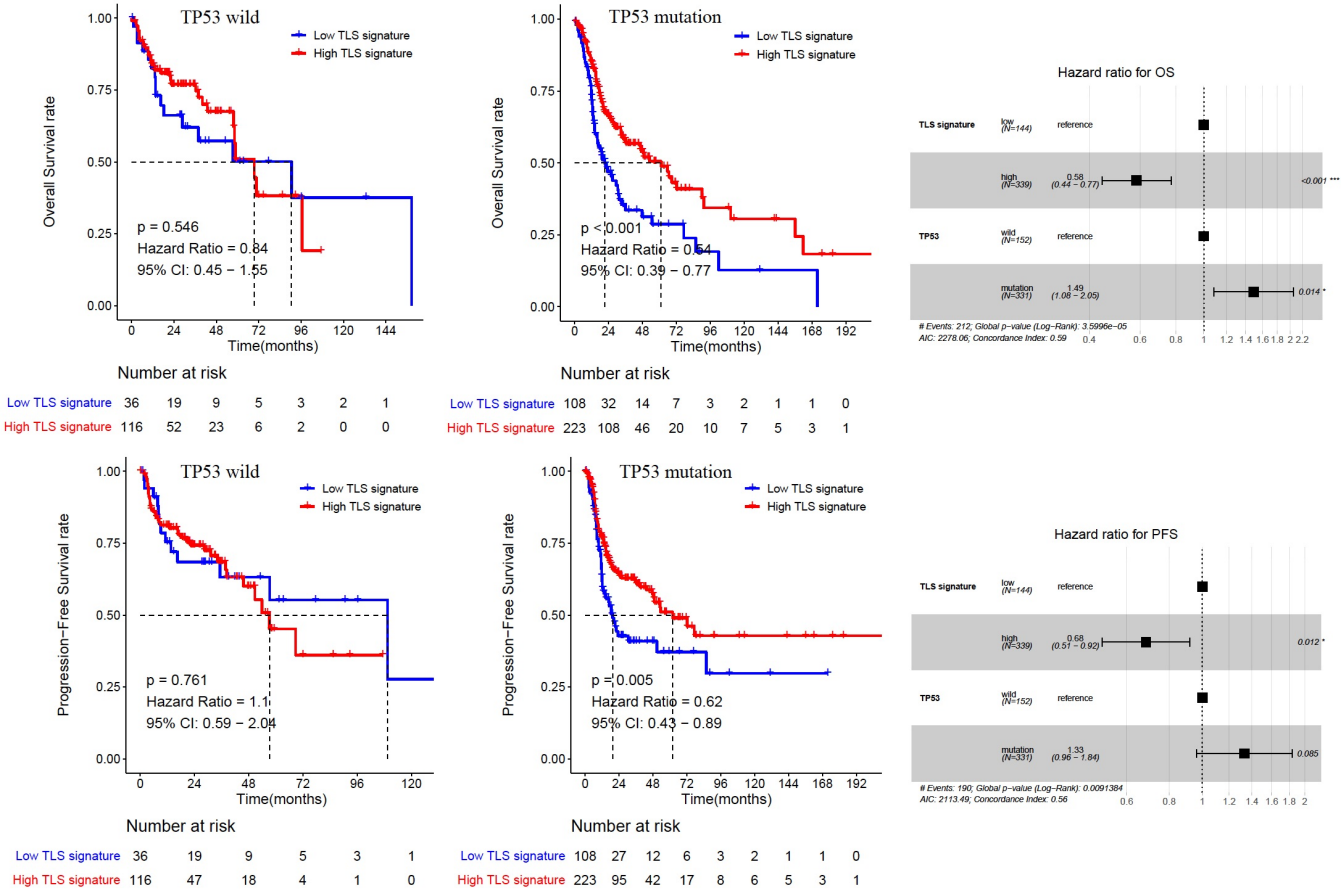
Differences in OS and PFS between groups with high and low TLS signatures in TP53 mutant and wild-type patients.

During tumorigenesis, driver gene mutations can positively and selectively drive normal cells to transform into proliferating cancer cells. The panorama of gene mutations (mutation frequency >5%) in HNSCC patients is shown in Fig. (**4A**), among which TP53 mutations have the highest frequency. Meanwhile, we detected more driver genes with significantly different mutation frequencies in both groups with high and low TLS signatures (Fig. **4B**), and they were mainly enriched in NRF2 and cell cycle pathways (Fig. **4C**). Further survival analysis revealed that HNSCC patients with TP53 mutations in the high TLS signature group had superior OS (*P *< 0.001) and PFS (*P*=0.005) than those in the low group, while patients with TP53 wild-type had no discernible difference (Fig. **5**). Cox regression analysis indicates the interaction between TP53 and TLS signature (Fig. **5**).

### 3.5 Validation of the Impact of TLS Signature on the Prognosis and Immune Microenvironment of HNSCC Patients

**Fig. (6):**
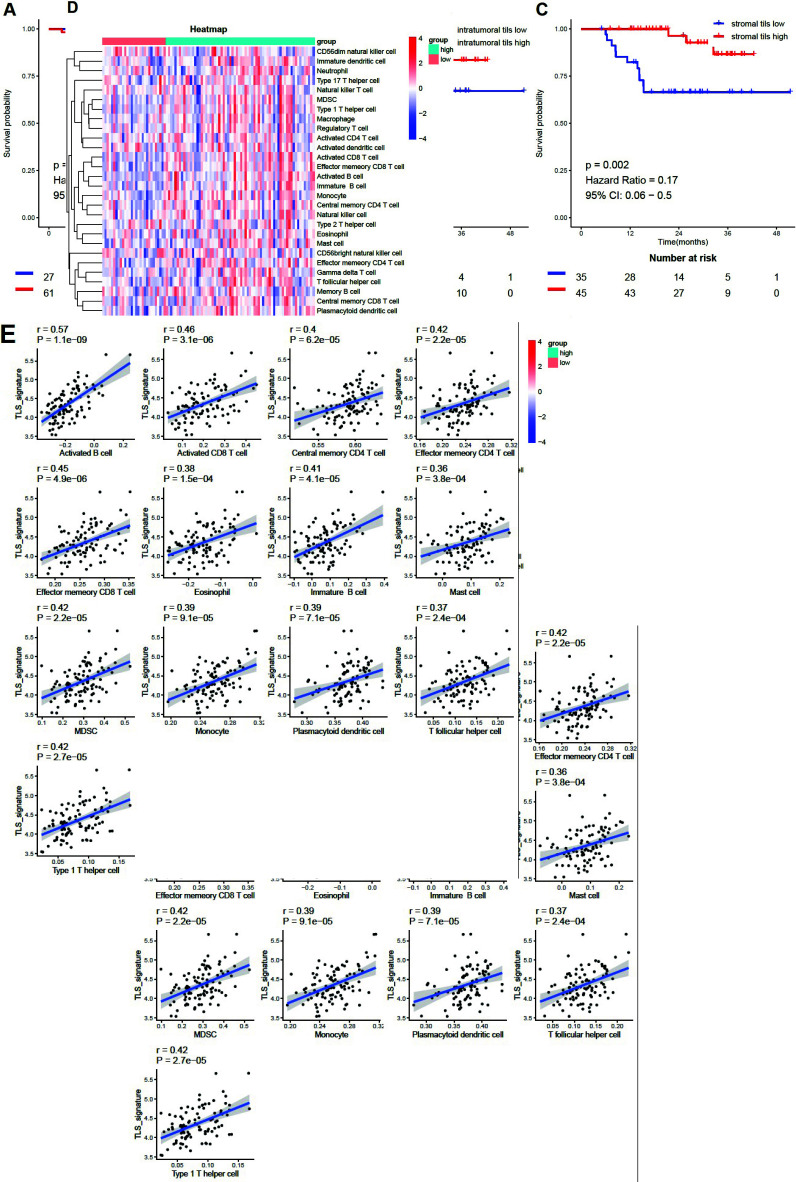
Validating the impact of TLS signatures on the prognosis and immune microenvironment of HNSCC patients. (**A**). Validate the differences in overall survival between groups with high and low TLS signatures in the GSE41613 dataset (n = 88). (**B** and **C**). Assess the prognostic implications of stromal and intratumoral TLS within the GSE41613 dataset (n = 80). (**D**). Differences in immune cell subpopulations between the groups with high TLS signature (n = 68) and low TLS signature (n = 29); (**E**). Correlation analysis of TLS with major immune cell subpopulations. *P *< 0.05 was considered a significant difference.

To reinforce the reliability of the aforementioned assay results, we conducted a validation study to confirm the impact of TLS signature on the immune microenvironment of HNSCC patients using the GSE41613 dataset. HNSCC patients were categorized into two groups based on their TLS signature levels: high or low (if lower than 30%). Fig. (**6A**) validates the predictive capacity of TLS signatures for patient prognosis (*P*=0.002). Figs. (**6B** and **C**) employ histopathologically assessed TLS structures, demonstrating that patients with high stroma TLS experience a better prognosis than those with low stroma TLS (*P*=0.002), whereas intratumoral TLS manifests no discernible impact on patient prognosis. Fig. (**6D**) demonstrates the association of high/low TLS signature with immune cell subpopulations. Consistent with the prior findings, the immune cell subpopulations in the group with high TLS signatures were significantly higher than those with low TLS signatures (*P *< 0.05, Fig. **S5**). Additionally, tumor-infiltrating immune cells displayed a noticeable and positive correlation with TLS signature (*P *< 0.001, Fig. **6E**).

### 3.6 Biological Processes and Signaling Pathways Linked to TLS Signature

**Fig. (7):**
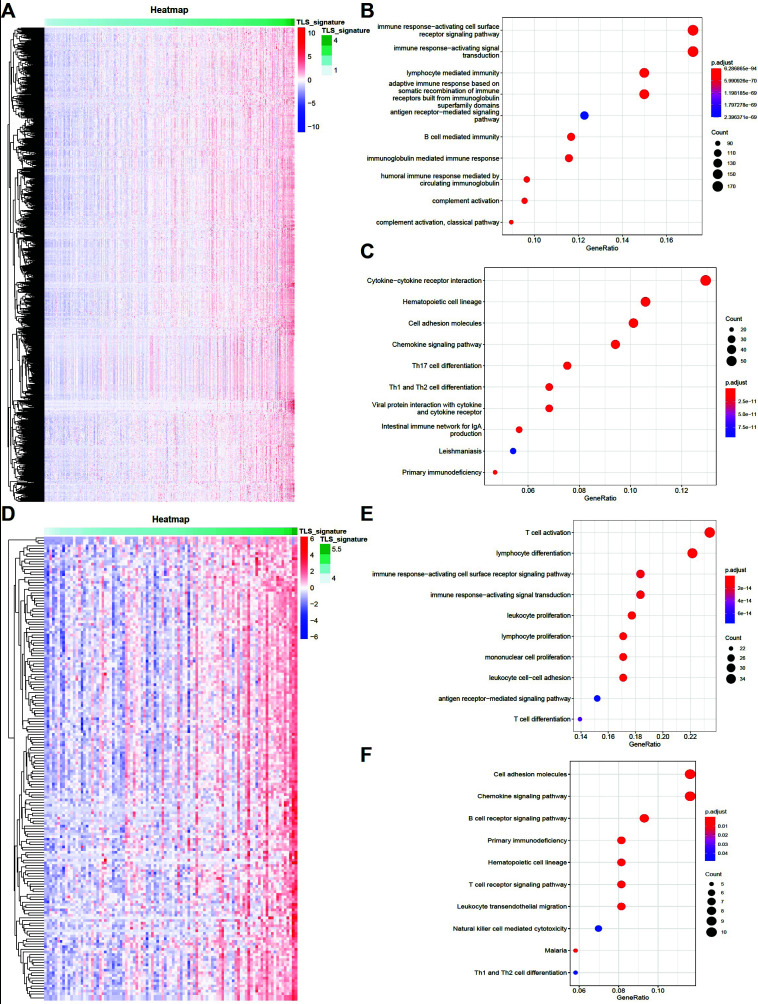
Biological processes and signaling pathways linked to TLS signature. (**A**). Genes with high correlation with TLS signature in the TCGA dataset; (**B**): GO enrichment analysis of genes with a correlation greater than 0.5 with the TLS signature in the TCGA dataset; (**C**): KEGG pathway analysis of genes with a correlation greater than 0.5 with the TLS signature in the TCGA dataset; (**D**). Genes with high correlation with TLS signature in the GSE41613 dataset; (**E**): GO enrichment analysis of genes with a correlation greater than 0.5 with TLS signature in the GSE41613 dataset; (**F**): KEGG pathway analysis of genes with a correlation greater than 0.5 with TLS signature in the GSE41613 dataset.

Finally, we explored the biological processes and signaling pathways that are significantly associated with TLS signature *via* analysis of the TCGA database and the GSE41613 dataset. As depicted in Figs. (**7A**-**7C**), in the TCGA dataset, the genes that correlated with TLS signature of more than 0.5 were primarily enriched in the cytokine receptor interaction pathway and were involved in the immune response activating cell surface receptor signaling pathway as well as immune response activating signal transduction. In the GSE41613 dataset, the genes that correlated with a TLS signature of more than 0.5 were predominantly involved in cell adhesion molecules and chemokine pathways, which were linked to T cell activation (Figs. **7D**-**F**). Moreover, in these datasets, immune-related biological processes and signaling pathways were significantly upregulated with an increase in the TLS signature.

## 4 DISCUSSION

TLS provides an important and localized microenvironment for cellular and humoral immunity. Given the relationship between TLS and clinical benefits in cancer patients, TLS may serve as a prognostic factor and a predictor [35, 36]. Our findings demonstrate that patients with elevated TLS feature scores exhibit improved survival rates and heightened infiltration of immune cell subsets. This association was consistently validated across independent cohorts, emphasizing the robustness of our results. In alignment with our findings, the presence of TLS has also been identified as a favorable prognostic factor for HNSCC and to promote immune therapeutic responses, which has also been confirmed in other cancers [17, 19, 27, 37].

A deeper exploration of the correlation between TLS signatures and the immune microenvironment revealed significant elevations in immune cell subsets and immune cycle characteristics (IGS1, IGS3, IGS4, IGS6, and IGS7) among patients with high TLS signatures. The positive correlation observed between these factors suggests a potential mechanism, where immunogenic cell death contributes to the release of molecules associated with injury [38]. The intense infiltration of regulatory T cells and high expression of checkpoint molecules on T cell subpopulations are distinctive features of the tumor microenvironment in HNSCC [39]. In 2013, Chen *et al*. introduced the concept of tumor immune circulation, revealing the mechanisms by which the immune system kills tumor cells [40]. The basic steps of the cancer immune cycle occur in the tumor itself and regional lymph nodes, with immune cells moving between these different sites. Anticancer immunotherapy aims to reactivate all steps of this cycle, including immunogenic cell death, T- cell initiation and activation, and enhancement of effector T- cell activity [41]. Our findings lead us to conclude that patients with a high TLS signature have better immunotherapeutic outcomes. These results offer new perspectives on the development of innovative prognostic markers and immunotherapeutic approaches for HNSCC.

In considering the genetic landscape of HNSCC, driver gene mutations, including frequently observed mutations in MYC, APC, TP53, and KRAS [42], play a pivotal role in shaping cancer immune phenotypes and tolerance [43]. TP53, with the highest mutation frequency in our study, emerges as a potential biomarker for prognosis in HNSCC patients. Stratifying patients based on TP53 mutation status, in conjunction with TLS signature analysis, may enhance the accuracy of prognostic stratification, which is consistent with previous research [44, 45]. A recent study showed that lung adenocarcinoma patients with TP53 mutation had a good OS and high TLS signature, while poorer OS was associated with a lower TLS signature [46]. In our study, there were notable differences in the mutation status and mutation frequency of driver genes between TLS signature high/low groups. Therefore, an integrated evaluation of driver genes of TLS signature and mutation status would be helpful for immunotherapy in HNSCC patients.

Our study also delves into the biological processes and signaling pathways associated with TLS signatures, revealing significant activation of immune-related processes as TLS signatures increase. This suggests a potential role for TLS signatures in regulating and developing immune responses in HNSCC. The study on co-stimulatory molecules, particularly PD-1 and its ligands PD-L1 and PD-L2, provides insights into immune system evasion mechanisms [47]. ICI represents a revolutionary breakthrough in cancer therapy, wherein the PD-1/PD-L1 axis plays a direct role in cancer immune modulation and is considered among the most pertinent immune checkpoint inhibitors [8]. The expression of PD-L1 is positively correlated with the efficacy of immunotherapy [5, 12, 13]. Several studies underscore challenges associated with PD-L1 assessment, including tumor heterogeneity, sample variability, inter-observer differences, and disparities in clones and platforms utilized for analysis [48, 49]. We conducted an analysis of the expression of immune checkpoint-related genes in different TLS features of HNSCC patients in the TCGA database, including PD-1 (encoded by PDCD1) and PD-L1 (encoded by CD274), revealing increased expression of PDCD1 and CD274 in high TLS patients. This finding aligns with prior cancer research, and a systematic review of the literature indicates that the sarcoma subtype with high tertiary lymphoid structures often responds to ICI. However, further investigation is warranted [50]. An analysis by Lin *et al*. of TLS signature expression and cellular composition of the tumor immune microenvironment suggested that TLS signature scores are strongly correlated with the level of T cell infiltration [51]. This finding is consistent with the results obtained from our analysis of GSE41613, which suggests that TLS signature can predict the therapeutic response or survival outcome of immunotherapy in HNSCC patients. This study has some limitations. For instance, all analyzed samples were derived from public datasets, warranting further validation. Consequently, future research necessitates more extensive clinical trials to substantiate our hypotheses, thereby offering novel insights into immunotherapy for HNSCC patients.

## Figures and Tables

**Fig. (1):**
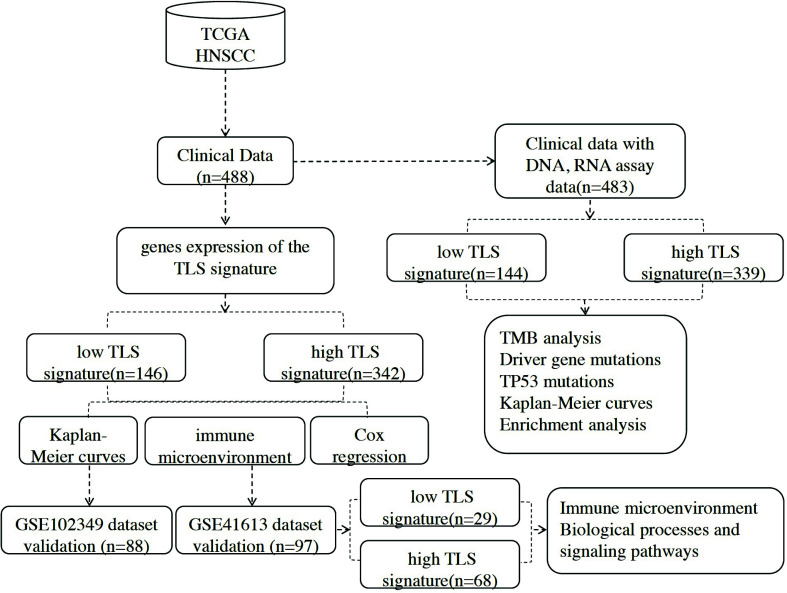
Flowchart showing the analysis process.

**Table 1:** Basic clinical information for patients with HNSCC (n=483).

**-**	**TLS Signature High n=339**	**TLS Signature Low n=144**	***P* Value**
**Gender**	-	-	<0.001
**Female**	70 (20.6%)	61(42.4%)	-
**Male**	269(79.4%)	83(57.6%)	-
**Age, mean (SD)**	61.1(11.5)	61.2(12.1)	0.913
**Clinical stage**	-	-	0.169
**Stage I-II**	84(25.5%)	27(19.1%)	-
**Stage III-IV**	245(74.5%)	114(80.9%)	-
**Histological grade**	-	-	0.043
**G1-2**	35(10.5%)	25(17.7%)	-
**G3-4**	299(89.5%)	116(82.3%)	-
**TP53**	-	-	0.059
**Mutation**	223(65.8%)	108(75.0%)	-
**Wild**	116(34.2%)	36(25.0%)	-

**Abbreviation:** TLS: tertiary lymphoid structures

**Table 2:** Univariate and multivariate analyses (n = 488).

**-**	**Univariate Analysis**		**Multivariate Analysis**	
**-**	**HR (95%CI)**	***P* Value**	**HR (95%CI)**	***P* Value**
**Overall survival**	-	-	-	-
Age (>60 *vs*. ≤60)	1.21 (0.93-1.59)	0.161	-	-
Gender (Male *vs*. Female)	0.77 (0.58-1.03)	0.081	0.84 (0.62-1.15)	0.276
Race (White *vs*. Non-white)	0.70 (0.47-1.05)	0.082	0.65 (0.43-0.98)	0.040
Clinical stage (III-IV *vs*. I-II)	1.22 (0.88-1.69)	0.242	-	-
Histological grade (G3-4 *vs*. G1-2)	0.93 (0.69-1.26)	0.636	-	-
TLS signature (high *vs*. low)	0.59 (0.45-0.78)	<0.001	0.62 (0.47-0.84)	0.002
**Progression-free survival**	-	-	-	-
Age (>60 *vs*. ≤60)	1.03 (0.77-1.36)	0.866	-	-
Gender (Male *vs*. Female)	1.09 (0.78-1.51)	0.627	-	-
Race (White *vs*. Non-white)	0.72 (0.47-1.09)	0.122	-	-
Clinical stage (III-IV *vs*. I-II)	1.21(0.85-1.71)	0.298	-	-
Histological grade (G3-4 *vs*. G1-2)	0.92 (0.66-1.27)	0.595	-	-
TLS signature (high *vs*. low)	0.69 (0.51-0.93)	0.014	0.69 (0.51-0.93)	0.014

**Abbreviation:** TLS: tertiary lymphoid structures.

## Data Availability

The datasets used during the current study are available from the corresponding author upon reasonable request.

## LIST OF ABBREVIATIONS

FDR: False Discovery Rate
HNSCC: Head and Neck Squamous Cell Carcinoma
IGS: Immunogram Score
IQR: Interquartile Range
KEGG: Kyoto Gene and Genome Encyclopedia
OS: Overall Survival
PFS: Progression-free Survival
SNV: Single Nucleotide Variant
ssGSEA: Single-sample Gene Set Enrichment Analysis
TCGA: The Cancer Genome Atlas
TIL: Tumor-infiltrating
TIL-B: Tumor-infiltrating B Cells
TLS: Tertiary Lymphoid Structures

## References

[1] Mandal R., Şenbabaoğlu Y., Desrichard A., Havel J.J., Dalin M.G., Riaz N., Lee K.W., Ganly I., Hakimi A.A., Chan T.A., Morris L.G.T. (2016). The head and neck cancer immune landscape and its immunotherapeutic implications.. JCI Insight.

[2] Chi A.C., Day T.A., Neville B.W. (2015). Oral cavity and oropharyngeal squamous cell carcinoma—an update.. CA Cancer J. Clin..

[3] Johnson D.E., Burtness B., Leemans C.R., Lui V.W.Y., Bauman J.E., Grandis J.R. (2020). Head and neck squamous cell carcinoma.. Nat. Rev. Dis. Primers.

[4] Sung H., Ferlay J., Siegel R.L., Laversanne M., Soerjomataram I., Jemal A., Bray F. (2021). Global cancer statistics 2020: Globocan estimates of incidence and mortality worldwide for 36 cancers in 185 countries.. CA Cancer J. Clin..

[5] Burtness B., Harrington K.J., Greil R., Soulières D., Tahara M., de Castro G., Psyrri A., Basté N., Neupane P., Bratland Å., Fuereder T., Hughes B.G.M., Mesía R., Ngamphaiboon N., Rordorf T., Wan Ishak W.Z., Hong R.L., González Mendoza R., Roy A., Zhang Y., Gumuscu B., Cheng J.D., Jin F., Rischin D., Lerzo G., Tatangelo M., Varela M., Zarba J.J., Boyer M., Gan H., Gao B., Hughes B., Mallesara G., Rischin D., Taylor A., Burian M., Fuereder T., Greil R., Barrios C.H., de Castro Junior D.O., Castro G., Franke F.A., Girotto G., Lima I.P.F., Nicolau U.R., Pinto G.D.J., Santos L., Victorino A-P., Chua N., Couture F., Gregg R., Hansen A., Hilton J., McCarthy J., Soulieres D., Ascui R., Gonzalez P., Villanueva L., Torregroza M., Zambrano A., Holeckova P., Kral Z., Melichar B., Prausova J., Vosmik M., Andersen M., Gyldenkerne N., Jurgens H., Putnik K., Reinikainen P., Gruenwald V., Laban S., Aravantinos G., Boukovinas I., Georgoulias V., Psyrri A., Kwong D., Al-Farhat Y., Csoszi T., Erfan J., Horvai G., Landherr L., Remenar E., Ruzsa A., Szota J., Billan S., Gluck I., Gutfeld O., Popovtzer A., Benasso M., Bui S., Ferrari V., Licitra L., Nole F., Fujii T., Fujimoto Y., Hanai N., Hara H., Matsumoto K., Mitsugi K., Monden N., Nakayama M., Okami K., Oridate N., Shiga K., Shimizu Y., Sugasawa M., Tahara M., Takahashi M., Takahashi S., Tanaka K., Ueda T., Yamaguchi H., Yamazaki T., Yasumatsu R., Yokota T., Yoshizaki T., Kudaba I., Stara Z., Wan Ishak W.Z., Cheah S.K., Aguilar Ponce J., Gonzalez Mendoza R., Hernandez Hernandez C., Medina Soto F., Buter J., Hoeben A., Oosting S., Suijkerbuijk K., Bratland A., Brydoey M., Alvarez R., Mas L., Caguioa P., Querol J., Regala E.E., Tamayo M.B., Villegas E.M., Kawecki A., Karpenko A., Klochikhin A., Smolin A., Zarubenkov O., Goh B.C., Cohen G., du Toit J., Jordaan C., Landers G., Ruff P., Szpak W., Tabane N., Brana I., Iglesias Docampo L., Lavernia J., Mesia R., Abel E., Muratidu V., Nielsen N., Cristina V., Rordorf T., Rothschild S., Hong R-L., Wang H-M., Yang M-H., Yeh S-P., Yen C-J., Ngamphaiboon N., Soparattanapaisarn N., Sriuranpong V., Aksoy S., Cicin I., Ekenel M., Harputluoglu H., Ozyilkan O., Harrington K., Agarwala S., Ali H., Alter R., Anderson D., Bruce J., Burtness B., Campbell N., Conde M., Deeken J., Edenfield W., Feldman L., Gaughan E., Goueli B., Halmos B., Hegde U., Hunis B., Jotte R., Karnad A., Khan S., Laudi N., Laux D., Martincic D., McCune S., McGaughey D., Misiukiewicz K., Mulford D., Nadler E., Neupane P., Nunnink J., Ohr J., O’Malley M., Patson B., Paul D., Popa E., Powell S., Redman R., Rella V., Rocha Lima C., Sivapiragasam A., Su Y., Sukari A., Wong S., Yilmaz E., Yorio J., KEYNOTE-048 Investigators (2019). Pembrolizumab alone or with chemotherapy *versus* cetuximab with chemotherapy for recurrent or metastatic squamous cell carcinoma of the head and neck (KEYNOTE-048): A randomised, open-label, phase 3 study.. Lancet.

[6] Gomes-da-Silva L.C., Kepp O., Kroemer G. (2020). Regulatory approval of photoimmunotherapy: Photodynamic therapy that induces immunogenic cell death.. OncoImmunology.

[7] Amaria R.N., Prieto P.A., Tetzlaff M.T., Reuben A., Andrews M.C., Ross M.I., Glitza I.C., Cormier J., Hwu W.J., Tawbi H.A., Patel S.P., Lee J.E., Gershenwald J.E., Spencer C.N., Gopalakrishnan V., Bassett R., Simpson L., Mouton R., Hudgens C.W., Zhao L., Zhu H., Cooper Z.A., Wani K., Lazar A., Hwu P., Diab A., Wong M.K., McQuade J.L., Royal R., Lucci A., Burton E.M., Reddy S., Sharma P., Allison J., Futreal P.A., Woodman S.E., Davies M.A., Wargo J.A. (2018). Neoadjuvant plus adjuvant dabrafenib and trametinib *versus* standard of care in patients with high-risk, surgically resectable melanoma: A single-centre, open-label, randomised, phase 2 trial.. Lancet Oncol..

[8] Wusiman D., Li W., Guo L., Huang Z., Zhang Y., Zhang X., Zhao X., Li L., An Z., Li Z., Ying J., An C. (2023). Comprehensive analysis of single-cell and bulk RNA-sequencing data identifies B cell marker genes signature that predicts prognosis and analysis of immune checkpoints expression in head and neck squamous cell carcinoma.. Heliyon.

[9] Wu X., Gu Z., Chen Y., Chen B., Chen W., Weng L., Liu X. (2019). Application of PD-1 blockade in cancer immunotherapy.. Comput. Struct. Biotechnol. J..

[10] Gavrielatou N., Fortis E., Spathis A., Anastasiou M., Economopoulou P., Foukas G.R.P., Lelegiannis I.M., Rusakiewicz S., Vathiotis I., Aung T.N., Tissot S., Kastrinou A., Kotsantis I., Vagia E.M., Panayiotides I., Rimm D.L., Coukos G., Homicsko K., Foukas P., Psyrri A. (2023). B-cell infiltration is associated with survival outcomes following programmed cell death protein 1 inhibition in head and neck squamous cell carcinoma.. Ann. Oncol..

[11] Borel C., Jung A.C., Burgy M. (2020). Immunotherapy breakthroughs in the treatment of recurrent or metastatic head and neck squamous cell carcinoma.. Cancers.

[12] Ferris R.L., Blumenschein G., Fayette J., Guigay J., Colevas A.D., Licitra L., Harrington K., Kasper S., Vokes E.E., Even C., Worden F., Saba N.F., Iglesias Docampo L.C., Haddad R., Rordorf T., Kiyota N., Tahara M., Monga M., Lynch M., Geese W.J., Kopit J., Shaw J.W., Gillison M.L. (2016). Nivolumab for recurrent squamous-cell carcinoma of the head and neck.. N. Engl. J. Med..

[13] Seiwert T.Y., Burtness B., Mehra R., Weiss J., Berger R., Eder J.P., Heath K., McClanahan T., Lunceford J., Gause C., Cheng J.D., Chow L.Q. (2016). Safety and clinical activity of pembrolizumab for treatment of recurrent or metastatic squamous cell carcinoma of the head and neck (KEYNOTE-012): An open-label, multicentre, phase 1b trial.. Lancet Oncol..

[14] Ruffin A.T., Cillo A.R., Tabib T., Liu A., Onkar S., Kunning S.R., Lampenfeld C., Atiya H.I., Abecassis I., Kürten C.H.L., Qi Z., Soose R., Duvvuri U., Kim S., Oesterrich S., Lafyatis R., Coffman L.G., Ferris R.L., Vignali D.A.A., Bruno T.C. (2021). B cell signatures and tertiary lymphoid structures contribute to outcome in head and neck squamous cell carcinoma.. Nat. Commun..

[15] Economopoulou P., Kotsantis I., Psyrri A. (2021). B cells and their role in shaping the immune response in squamous cell carcinoma of the head and neck.. Immunotherapy.

[16] Cabrita R., Lauss M., Sanna A., Donia M., Skaarup Larsen M., Mitra S., Johansson I., Phung B., Harbst K., Vallon-Christersson J., van Schoiack A., Lövgren K., Warren S., Jirström K., Olsson H., Pietras K., Ingvar C., Isaksson K., Schadendorf D., Schmidt H., Bastholt L., Carneiro A., Wargo J.A., Svane I.M., Jönsson G. (2020). Tertiary lymphoid structures improve immunotherapy and survival in melanoma.. Nature.

[17] Helmink B.A., Reddy S.M., Gao J., Zhang S., Basar R., Thakur R., Yizhak K., Sade-Feldman M., Blando J., Han G., Gopalakrishnan V., Xi Y., Zhao H., Amaria R.N., Tawbi H.A., Cogdill A.P., Liu W., LeBleu V.S., Kugeratski F.G., Patel S., Davies M.A., Hwu P., Lee J.E., Gershenwald J.E., Lucci A., Arora R., Woodman S., Keung E.Z., Gaudreau P.O., Reuben A., Spencer C.N., Burton E.M., Haydu L.E., Lazar A.J., Zapassodi R., Hudgens C.W., Ledesma D.A., Ong S., Bailey M., Warren S., Rao D., Krijgsman O., Rozeman E.A., Peeper D., Blank C.U., Schumacher T.N., Butterfield L.H., Zelazowska M.A., McBride K.M., Kalluri R., Allison J., Petitprez F., Fridman W.H., Sautès-Fridman C., Hacohen N., Rezvani K., Sharma P., Tetzlaff M.T., Wang L., Wargo J.A. (2020). B cells and tertiary lymphoid structures promote immunotherapy response.. Nature.

[18] Wu Z., Zhou J., Xiao Y., Ming J., Zhou J., Dong F., Zhou X., Xu Z., Zhao X., Lei P., Huang T. (2022). CD20^+^CD22^+^ADAM28^+^ B cells in tertiary lymphoid structures promote immunotherapy response.. Front. Immunol..

[19] Petitprez F., de Reyniès A., Keung E.Z., Chen T.W.W., Sun C.M., Calderaro J., Jeng Y.M., Hsiao L.P., Lacroix L., Bougoüin A., Moreira M., Lacroix G., Natario I., Adam J., Lucchesi C., Laizet Y., Toulmonde M., Burgess M.A., Bolejack V., Reinke D., Wani K.M., Wang W.L., Lazar A.J., Roland C.L., Wargo J.A., Italiano A., Sautès-Fridman C., Tawbi H.A., Fridman W.H. (2020). B cells are associated with survival and immunotherapy response in sarcoma.. Nature.

[20] Dieu-Nosjean M.C., Giraldo N.A., Kaplon H., Germain C., Fridman W.H., Sautès-Fridman C. (2016). Tertiary lymphoid structures, drivers of the anti‐tumor responses in human cancers.. Immunol. Rev..

[21] Tertiary lymphoid structures validated as biomarker. (2023). Cancer Discov..

[22] Wang X., Juncker-Jensen A., Huang G., Nagy M.L., Lu X., Cheng L., Lu X. (2023). Spatial relationship of tertiary lymphoid structures and tumor‐associated neutrophils in bladder cancer and prognostic potential for anti‐PD‐L1 immunotherapy.. Cancer Commun..

[23] Calderaro J., Petitprez F., Becht E., Laurent A., Hirsch T.Z., Rousseau B., Luciani A., Amaddeo G., Derman J., Charpy C., Zucman-Rossi J., Fridman W.H., Sautès-Fridman C. (2019). Intra-tumoral tertiary lymphoid structures are associated with a low risk of early recurrence of hepatocellular carcinoma.. J. Hepatol..

[24] Zhou L., Xu B., Liu Y., Wang Z. (2021). Tertiary lymphoid structure signatures are associated with survival and immunotherapy response in muscle-invasive bladder cancer.. OncoImmunology.

[25] Wood O., Woo J., Seumois G., Savelyeva N., McCann K.J., Singh D., Jones T., Peel L., Breen M.S., Ward M., Garrido Martin E., Sanchez-Elsner T., Thomas G., Vijayanand P., Woelk C.H., King E., Ottensmeier C., Consortium S., SPARC Consortium (2016). Gene expression analysis of TIL rich HPV-driven head and neck tumors reveals a distinct B-cell signature when compared to HPV independent tumors.. Oncotarget.

[26] Schinke H., Shi E., Lin Z., Quadt T., Kranz G., Zhou J., Wang H., Hess J., Heuer S., Belka C., Zitzelsberger H., Schumacher U., Genduso S., Riecken K., Gao Y., Wu Z., Reichel C.A., Walz C., Canis M., Unger K., Baumeister P., Pan M., Gires O. (2022). A transcriptomic map of EGFR-induced epithelial-to-mesenchymal transition identifies prognostic and therapeutic targets for head and neck cancer.. Mol. Cancer.

[27] Li H., Zhu S.W., Zhou J.J., Chen D.R., Liu J., Wu Z.Z., Wang W.Y., Zhang M.J., Sun Z.J. (2023). Tertiary lymphoid structure raises survival and immunotherapy in HPV ^−^ HNSCC.. J. Dent. Res..

[28] Shen A., Ye Y., Chen F., Xu Y., Zhang Z., Zhao Q., Zeng Z. (2022). Integrated multi-omics analysis identifies CD73 as a prognostic biomarker and immunotherapy response predictor in head and neck squamous cell carcinoma.. Front. Immunol..

[29] Chen Y., Li Z.Y., Zhou G.Q., Sun Y. (2021). An immune-related gene prognostic index for head and neck squamous cell carcinoma.. Clin. Cancer Res..

[30] Thorsson V., Gibbs D.L., Brown S.D., Wolf D., Bortone D.S., Ou Yang T.H., Porta-Pardo E., Gao G.F., Plaisier C.L., Eddy J.A., Ziv E., Culhane A.C., Paull E.O., Sivakumar I.K.A., Gentles A.J., Malhotra R., Farshidfar F., Colaprico A., Parker J.S., Mose L.E., Vo N.S., Liu J., Liu Y., Rader J., Dhankani V., Reynolds S.M., Bowlby R., Califano A., Cherniack A.D., Anastassiou D., Bedognetti D., Mokrab Y., Newman A.M., Rao A., Chen K., Krasnitz A., Hu H., Malta T.M., Noushmehr H., Pedamallu C.S., Bullman S., Ojesina A.I., Lamb A., Zhou W., Shen H., Choueiri T.K., Weinstein J.N., Guinney J., Saltz J., Holt R.A., Rabkin C.S., Lazar A.J., Serody J.S., Demicco E.G., Disis M.L., Vincent B.G., Shmulevich I., Caesar-Johnson S.J., Demchok J.A., Felau I., Kasapi M., Ferguson M.L., Hutter C.M., Sofia H.J., Tarnuzzer R., Wang Z., Yang L., Zenklusen J.C., Zhang J.J., Chudamani S., Liu J., Lolla L., Naresh R., Pihl T., Sun Q., Wan Y., Wu Y., Cho J., DeFreitas T., Frazer S., Gehlenborg N., Getz G., Heiman D.I., Kim J., Lawrence M.S., Lin P., Meier S., Noble M.S., Saksena G., Voet D., Zhang H., Bernard B., Chambwe N., Dhankani V., Knijnenburg T., Kramer R., Leinonen K., Liu Y., Miller M., Reynolds S., Shmulevich I., Thorsson V., Zhang W., Akbani R., Broom B.M., Hegde A.M., Ju Z., Kanchi R.S., Korkut A., Li J., Liang H., Ling S., Liu W., Lu Y., Mills G.B., Ng K-S., Rao A., Ryan M., Wang J., Weinstein J.N., Zhang J., Abeshouse A., Armenia J., Chakravarty D., Chatila W.K., de Bruijn I., Gao J., Gross B.E., Heins Z.J., Kundra R., La K., Ladanyi M., Luna A., Nissan M.G., Ochoa A., Phillips S.M., Reznik E., Sanchez-Vega F., Sander C., Schultz N., Sheridan R., Sumer S.O., Sun Y., Taylor B.S., Wang J., Zhang H., Anur P., Peto M., Spellman P., Benz C., Stuart J.M., Wong C.K., Yau C., Hayes D.N., Parker J.S., Wilkerson M.D., Ally A., Balasundaram M., Bowlby R., Brooks D., Carlsen R., Chuah E., Dhalla N., Holt R., Jones S.J.M., Kasaian K., Lee D., Ma Y., Marra M.A., Mayo M., Moore R.A., Mungall A.J., Mungall K., Robertson A.G., Sadeghi S., Schein J.E., Sipahimalani P., Tam A., Thiessen N., Tse K., Wong T., Berger A.C., Beroukhim R., Cherniack A.D., Cibulskis C., Gabriel S.B., Gao G.F., Ha G., Meyerson M., Schumacher S.E., Shih J., Kucherlapati M.H., Kucherlapati R.S., Baylin S., Cope L., Danilova L., Bootwalla M.S., Lai P.H., Maglinte D.T., Van Den Berg D.J., Weisenberger D.J., Auman J.T., Balu S., Bodenheimer T., Fan C., Hoadley K.A., Hoyle A.P., Jefferys S.R., Jones C.D., Meng S., Mieczkowski P.A., Mose L.E., Perou A.H., Perou C.M., Roach J., Shi Y., Simons J.V., Skelly T., Soloway M.G., Tan D., Veluvolu U., Fan H., Hinoue T., Laird P.W., Shen H., Zhou W., Bellair M., Chang K., Covington K., Creighton C.J., Dinh H., Doddapaneni H.V., Donehower L.A., Drummond J., Gibbs R.A., Glenn R., Hale W., Han Y., Hu J., Korchina V., Lee S., Lewis L., Li W., Liu X., Morgan M., Morton D., Muzny D., Santibanez J., Sheth M., Shinbrot E., Wang L., Wang M., Wheeler D.A., Xi L., Zhao F., Hess J., Appelbaum E.L., Bailey M., Cordes M.G., Ding L., Fronick C.C., Fulton L.A., Fulton R.S., Kandoth C., Mardis E.R., McLellan M.D., Miller C.A., Schmidt H.K., Wilson R.K., Crain D., Curley E., Gardner J., Lau K., Mallery D., Morris S., Paulauskis J., Penny R., Shelton C., Shelton T., Sherman M., Thompson E., Yena P., Bowen J., Gastier-Foster J.M., Gerken M., Leraas K.M., Lichtenberg T.M., Ramirez N.C., Wise L., Zmuda E., Corcoran N., Costello T., Hovens C., Carvalho A.L., de Carvalho A.C., Fregnani J.H., Longatto-Filho A., Reis R.M., Scapulatempo-Neto C., Silveira H.C.S., Vidal D.O., Burnette A., Eschbacher J., Hermes B., Noss A., Singh R., Anderson M.L., Castro P.D., Ittmann M., Huntsman D., Kohl B., Le X., Thorp R., Andry C., Duffy E.R., Lyadov V., Paklina O., Setdikova G., Shabunin A., Tavobilov M., McPherson C., Warnick R., Berkowitz R., Cramer D., Feltmate C., Horowitz N., Kibel A., Muto M., Raut C.P., Malykh A., Barnholtz-Sloan J.S., Barrett W., Devine K., Fulop J., Ostrom Q.T., Shimmel K., Wolinsky Y., Sloan A.E., De Rose A., Giuliante F., Goodman M., Karlan B.Y., Hagedorn C.H., Eckman J., Harr J., Myers J., Tucker K., Zach L.A., Deyarmin B., Hu H., Kvecher L., Larson C., Mural R.J., Somiari S., Vicha A., Zelinka T., Bennett J., Iacocca M., Rabeno B., Swanson P., Latour M., Lacombe L., Têtu B., Bergeron A., McGraw M., Staugaitis S.M., Chabot J., Hibshoosh H., Sepulveda A., Su T., Wang T., Potapova O., Voronina O., Desjardins L., Mariani O., Roman-Roman S., Sastre X., Stern M-H., Cheng F., Signoretti S., Berchuck A., Bigner D., Lipp E., Marks J., McCall S., McLendon R., Secord A., Sharp A., Behera M., Brat D.J., Chen A., Delman K., Force S., Khuri F., Magliocca K., Maithel S., Olson J.J., Owonikoko T., Pickens A., Ramalingam S., Shin D.M., Sica G., Van Meir E.G., Zhang H., Eijckenboom W., Gillis A., Korpershoek E., Looijenga L., Oosterhuis W., Stoop H., van Kessel K.E., Zwarthoff E.C., Calatozzolo C., Cuppini L., Cuzzubbo S., DiMeco F., Finocchiaro G., Mattei L., Perin A., Pollo B., Chen C., Houck J., Lohavanichbutr P., Hartmann A., Stoehr C., Stoehr R., Taubert H., Wach S., Wullich B., Kycler W., Murawa D., Wiznerowicz M., Chung K., Edenfield W.J., Martin J., Baudin E., Bubley G., Bueno R., De Rienzo A., Richards W.G., Kalkanis S., Mikkelsen T., Noushmehr H., Scarpace L., Girard N., Aymerich M., Campo E., Giné E., Guillermo A.L., Van Bang N., Hanh P.T., Phu B.D., Tang Y., Colman H., Evason K., Dottino P.R., Martignetti J.A., Gabra H., Juhl H., Akeredolu T., Stepa S., Hoon D., Ahn K., Kang K.J., Beuschlein F., Breggia A., Birrer M., Bell D., Borad M., Bryce A.H., Castle E., Chandan V., Cheville J., Copland J.A., Farnell M., Flotte T., Giama N., Ho T., Kendrick M., Kocher J-P., Kopp K., Moser C., Nagorney D., O’Brien D., O’Neill B.P., Patel T., Petersen G., Que F., Rivera M., Roberts L., Smallridge R., Smyrk T., Stanton M., Thompson R.H., Torbenson M., Yang J.D., Zhang L., Brimo F., Ajani J.A., Gonzalez A.M.A., Behrens C., Bondaruk J., Broaddus R., Czerniak B., Esmaeli B., Fujimoto J., Gershenwald J., Guo C., Lazar A.J., Logothetis C., Meric-Bernstam F., Moran C., Ramondetta L., Rice D., Sood A., Tamboli P., Thompson T., Troncoso P., Tsao A., Wistuba I., Carter C., Haydu L., Hersey P., Jakrot V., Kakavand H., Kefford R., Lee K., Long G., Mann G., Quinn M., Saw R., Scolyer R., Shannon K., Spillane A., Stretch, Synott M., Thompson J., Wilmott J., Al-Ahmadie H., Chan T.A., Ghossein R., Gopalan A., Levine D.A., Reuter V., Singer S., Singh B., Tien N.V., Broudy T., Mirsaidi C., Nair P., Drwiega P., Miller J., Smith J., Zaren H., Park J-W., Hung N.P., Kebebew E., Linehan W.M., Metwalli A.R., Pacak K., Pinto P.A., Schiffman M., Schmidt L.S., Vocke C.D., Wentzensen N., Worrell R., Yang H., Moncrieff M., Goparaju C., Melamed J., Pass H., Botnariuc N., Caraman I., Cernat M., Chemencedji I., Clipca A., Doruc S., Gorincioi G., Mura S., Pirtac M., Stancul I., Tcaciuc D., Albert M., Alexopoulou I., Arnaout A., Bartlett J., Engel J., Gilbert S., Parfitt J., Sekhon H., Thomas G., Rassl D.M., Rintoul R.C., Bifulco C., Tamakawa R., Urba W., Hayward N., Timmers H., Antenucci A., Facciolo F., Grazi G., Marino M., Merola R., de Krijger R., Gimenez-Roqueplo A-P., Piché A., Chevalier S., McKercher G., Birsoy K., Barnett G., Brewer C., Farver C., Naska T., Pennell N.A., Raymond D., Schilero C., Smolenski K., Williams F., Morrison C., Borgia J.A., Liptay M.J., Pool M., Seder C.W., Junker K., Omberg L., Dinkin M., Manikhas G., Alvaro D., Bragazzi M.C., Cardinale V., Carpino G., Gaudio E., Chesla D., Cottingham S., Dubina M., Moiseenko F., Dhanasekaran R., Becker K-F., Janssen K-P., Slotta-Huspenina J., Abdel-Rahman M.H., Aziz D., Bell S., Cebulla C.M., Davis A., Duell R., Elder J.B., Hilty J., Kumar B., Lang J., Lehman N.L., Mandt R., Nguyen P., Pilarski R., Rai K., Schoenfield L., Senecal K., Wakely P., Hansen P., Lechan R., Powers J., Tischler A., Grizzle W.E., Sexton K.C., Kastl A., Henderson J., Porten S., Waldmann J., Fassnacht M., Asa S.L., Schadendorf D., Couce M., Graefen M., Huland H., Sauter G., Schlomm T., Simon R., Tennstedt P., Olabode O., Nelson M., Bathe O., Carroll P.R., Chan J.M., Disaia P., Glenn P., Kelley R.K., Landen C.N., Phillips J., Prados M., Simko J., Smith-McCune K., VandenBerg S., Roggin K., Fehrenbach A., Kendler A., Sifri S., Steele R., Jimeno A., Carey F., Forgie I., Mannelli M., Carney M., Hernandez B., Campos B., Herold-Mende C., Jungk C., Unterberg A., von Deimling A., Bossler A., Galbraith J., Jacobus L., Knudson M., Knutson T., Ma D., Milhem M., Sigmund R., Godwin A.K., Madan R., Rosenthal H.G., Adebamowo C., Adebamowo S.N., Boussioutas A., Beer D., Giordano T., Mes-Masson A-M., Saad F., Bocklage T., Landrum L., Mannel R., Moore K., Moxley K., Postier R., Walker J., Zuna R., Feldman M., Valdivieso F., Dhir R., Luketich J., Pinero E.M.M., Quintero-Aguilo M., Carlotti C.G., Dos Santos J.S., Kemp R., Sankarankuty A., Tirapelli D., Catto J., Agnew K., Swisher E., Creaney J., Robinson B., Shelley C.S., Godwin E.M., Kendall S., Shipman C., Bradford C., Carey T., Haddad A., Moyer J., Peterson L., Prince M., Rozek L., Wolf G., Bowman R., Fong K.M., Yang I., Korst R., Rathmell W.K., Fantacone-Campbell J.L., Hooke J.A., Kovatich A.J., Shriver C.D., DiPersio J., Drake B., Govindan R., Heath S., Ley T., Van Tine B., Westervelt P., Rubin M.A., Lee J.I., Aredes N.D., Mariamidze A., Cancer Genome Atlas Research Network (2018). The immune landscape of cancer.. Immunity.

[31] Wang Q., Zhao Y., Wang F., Tan G. (2022). A novel immune signature predicts immunotherapy responsiveness and reveals the landscape of the tumor immune microenvironment in head and neck squamous cell carcinoma.. Front. Genet..

[32] Charoentong P., Finotello F., Angelova M., Mayer C., Efremova M., Rieder D., Hackl H., Trajanoski Z. (2017). Pan-cancer immunogenomic analyses reveal genotype-immunophenotype relationships and predictors of response to checkpoint blockade.. Cell Rep..

[33] Angelova M., Charoentong P., Hackl H., Fischer M.L., Snajder R., Krogsdam A.M., Waldner M.J., Bindea G., Mlecnik B., Galon J., Trajanoski Z. (2015). Characterization of the immunophenotypes and antigenomes of colorectal cancers reveals distinct tumor escape mechanisms and novel targets for immunotherapy.. Genome Biol..

[34] Karasaki T., Nagayama K., Kuwano H., Nitadori J., Sato M., Anraku M., Hosoi A., Matsushita H., Morishita Y., Kashiwabara K., Takazawa M., Ohara O., Kakimi K., Nakajima J. (2017). An immunogram for the cancer-immunity cycle: Towards personalized immunotherapy of lung cancer.. J. Thorac. Oncol..

[35] Sautès-Fridman C., Petitprez F., Calderaro J., Fridman W.H. (2019). Tertiary lymphoid structures in the era of cancer immunotherapy.. Nat. Rev. Cancer.

[36] Teillaud J.L., Dieu-Nosjean M.C. (2017). Tertiary lymphoid structures: An anti-tumor school for adaptive immune cells and an antibody factory to fight cancer?. Front. Immunol..

[37] Le X., Ferrarotto R., Wise-Draper T., Gillison M. (2020). Evolving role of immunotherapy in recurrent metastatic head and neck cancer.. J. Natl. Compr. Canc. Netw..

[38] Lin W., Chen M., Hong L., Zhao H., Chen Q. (2018). Crosstalk between PD-1/PD-L1 blockade and its combinatorial therapies in tumor immune microenvironment: A focus on HNSCC.. Front. Oncol..

[39] Lechner A., Schlößer H., Rothschild S.I., Thelen M., Reuter S., Zentis P., Shimabukuro-Vornhagen A., Theurich S., Wennhold K., Garcia-Marquez M., Tharun L., Quaas A., Schauss A., Isensee J., Hucho T., Huebbers C., von Bergwelt-Baildon M., Beutner D. (2017). Characterization of tumor-associated T-lymphocyte subsets and immune checkpoint molecules in head and neck squamous cell carcinoma.. Oncotarget.

[40] Chen D.S., Mellman I. (2013). Oncology meets immunology: The cancer-immunity cycle.. Immunity.

[41] Pio R., Ajona D., Ortiz-Espinosa S., Mantovani A., Lambris J.D. (2019). Complementing the cancer-immunity cycle.. Front. Immunol..

[42] Brown A.L., Li M., Goncearenco A., Panchenko A.R. (2019). Finding driver mutations in cancer: Elucidating the role of background mutational processes.. PLOS Comput. Biol..

[43] Li J., Wang W., Zhang Y., Cieślik M., Guo J., Tan M., Green M.D., Wang W., Lin H., Li W., Wei S., Zhou J., Li G., Jing X., Vatan L., Zhao L., Bitler B., Zhang R., Cho K.R., Dou Y., Kryczek I., Chan T.A., Huntsman D., Chinnaiyan A.M., Zou W. (2020). Epigenetic driver mutations in ARID1A shape cancer immune phenotype and immunotherapy.. J. Clin. Invest..

[44] Leroy B., Anderson M., Soussi T. (2014). TP53 mutations in human cancer: Database reassessment and prospects for the next decade.. Hum. Mutat..

[45] Zhou G., Liu Z., Myers J.N. (2016). *TP53* mutations in head and neck squamous cell carcinoma and their impact on disease progression and treatment response.. J. Cell. Biochem..

[46] Feng H., Yang F., Qiao L., Zhou K., Wang J., Zhang J., Tian T., Du Y., Shangguan H. (2021). Prognostic significance of gene signature of tertiary lymphoid structures in patients with lung adenocarcinoma.. Front. Oncol..

[47] Outh-Gauer S., Morini A., Tartour E., Lépine C., Jung A.C., Badoual C. (2020). The microenvironment of head and neck cancers: Papillomavirus involvement and potential impact of immunomodulatory treatments.. Head Neck Pathol..

[48] de Ruiter E.J., Mulder F.J., Koomen B.M., Speel E.J., van den Hout M.F.C.M., de Roest R.H., Bloemena E., Devriese L.A., Willems S.M. (2021). Comparison of three PD-L1 immunohistochemical assays in head and neck squamous cell carcinoma (HNSCC).. Mod. Pathol..

[49] Marletta S., Fusco N., Munari E., Luchini C., Cimadamore A., Brunelli M., Querzoli G., Martini M., Vigliar E., Colombari R., Girolami I., Pagni F., Eccher A. (2022). Atlas of PD-L1 for pathologists: Indications, scores, diagnostic platforms and reporting systems.. J. Pers. Med..

[50] Anastasiou M., Kyriazoglou A., Kotsantis I., Economopoulou P., Kyrkasiadou M., Giannopoulou A., Kosmidou A., Smerdi D., Moutafi M., Gavrielatou N., Psyrri A. (2023). Immune checkpoint inhibitors in sarcomas: A systematic review.. Immuno-Oncology and Technology.

[51] Lin Z., Huang L., Li S., Gu J., Cui X., Zhou Y. (2020). Pan-cancer analysis of genomic properties and clinical outcome associated with tumor tertiary lymphoid structure.. Sci. Rep..

