# Supplementary material for: Tertiary Lymphoid Structures Gene Signature Predicts Prognosis and Immune Infiltration Analysis in Head and Neck Squamous Cell Carcinoma
Source: Curr Genomics. 2024 Jan 29;25(2):88–104. doi: 10.2174/0113892029278082240118053857 (PMC11092909; doi:10.2174/0113892029278082240118053857)
Supplement: Supplementary file 1 [file CG-25-88_SD1.pdf]

Supplementary Material

Tertiary Lymphoid Structures Gene Signature Predicts Prognosis and Immune Infiltration Analysis in Head and Neck Squamous Cell Carcinoma

Aiyan Xing[1,#], Dongxiao Lv[2,3,#], Changshun Wu[4,5], Kai Zhou[2,3], Tianhui Zhao[6], Lihua Zhao[6,*], Huaqing Wang[7,*] and Hong Feng[2,3,*]

[1]Department of Pathology, Shandong University Qilu Hospital, Jinan, Shandong 250012, China; [2]Cancer Center, Shandong Provincial Hospital, Cheeloo College of Medicine, Shandong University, Jinan, Shandong 250021, China; [3]Cancer Center, Shandong Provincial Hospital Affiliated to Shandong First Medical University, Jinan, Shandong 250021, China; [4]Department of Surgery, Shandong Provincial Hospital, Cheeloo College of Medicine, Shandong University, Jinan, Shandong 250021, China; [5]Department of Surgery, Shandong Provincial Hospital Affiliated to Shandong First Medical University, Jinan, Shandong 250021, China; [6]Department of Translational Medicine, Genecast Biotechnology Co., Ltd, Wuxi, Jiangsu 214104, China; [7]Department of Medical Oncology, Tianjin Union Medical Center, The Affiliated Hospital of Nankai University, Tianjin 300000, China

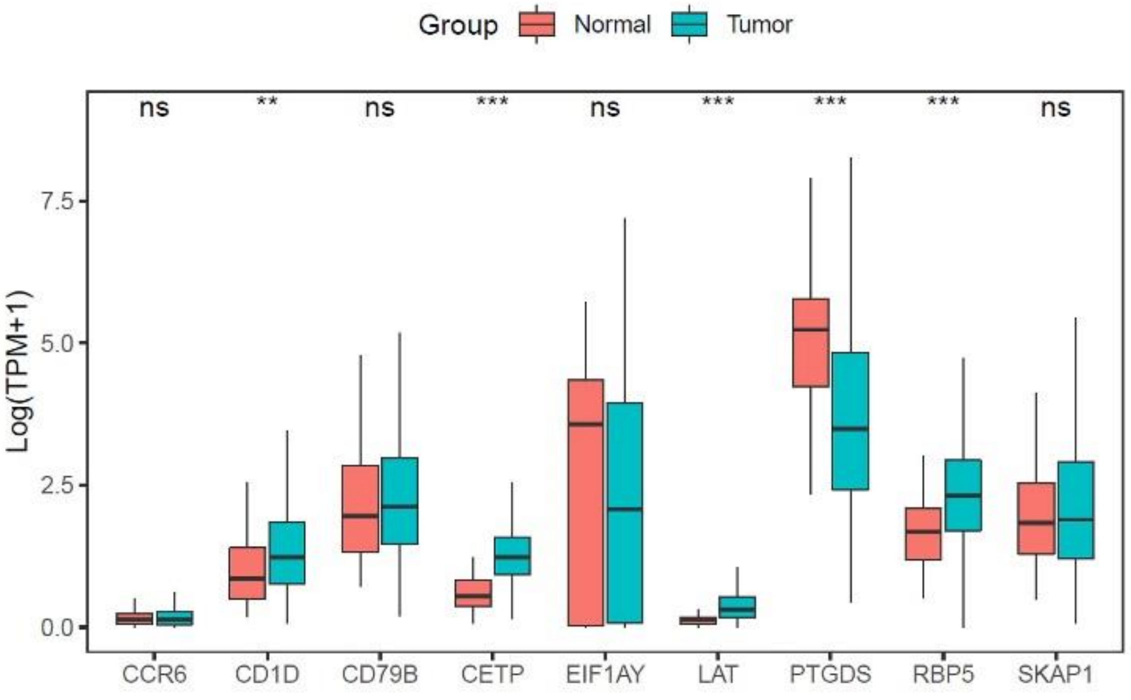

Fig. (S1). Differences in 9 genes expression contained in the TLS signature in tumor tissues *versus* normal tissues.

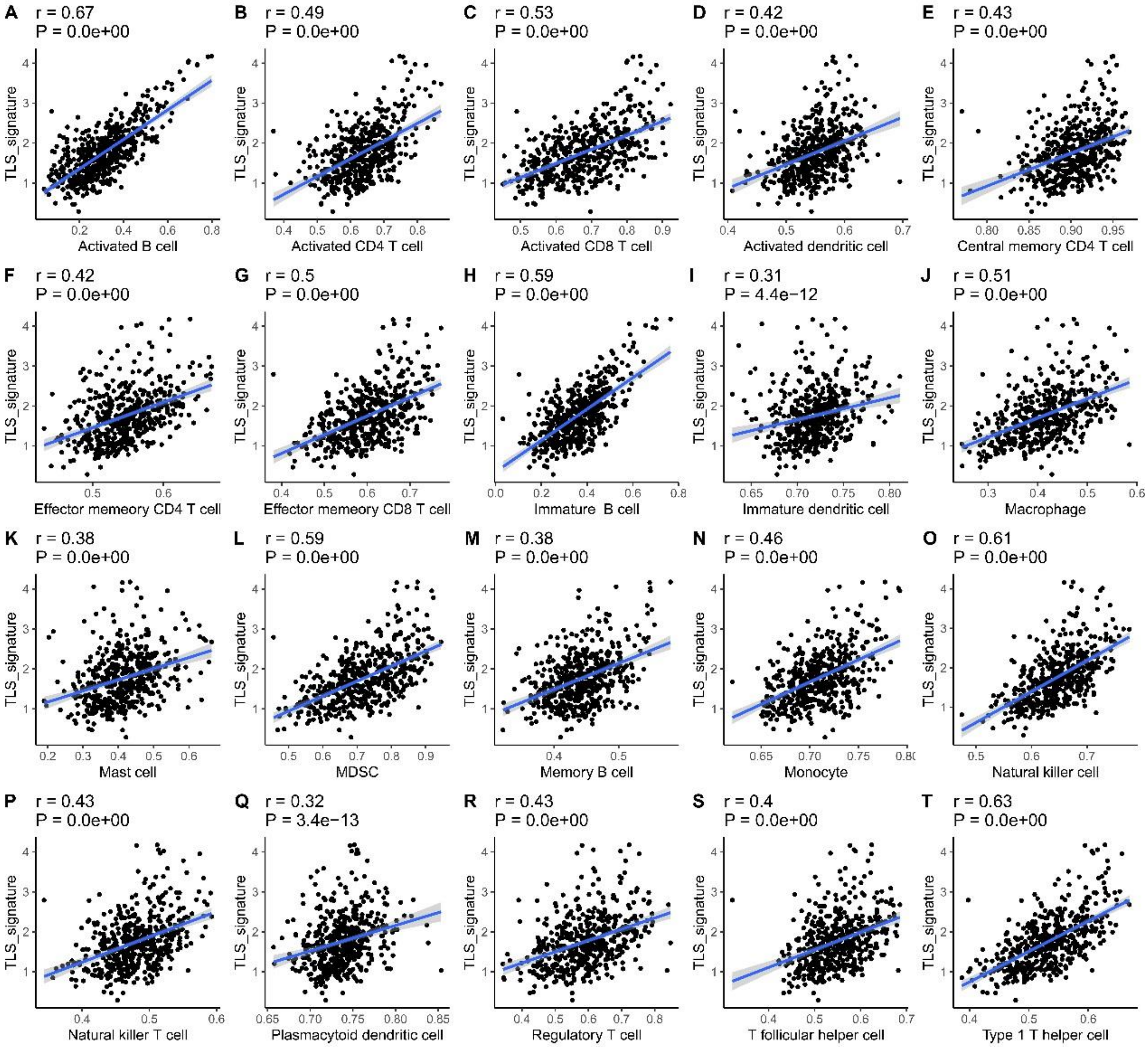

Fig. (S2). Correlation of TLS major component immune cell subsets with TLS signature.

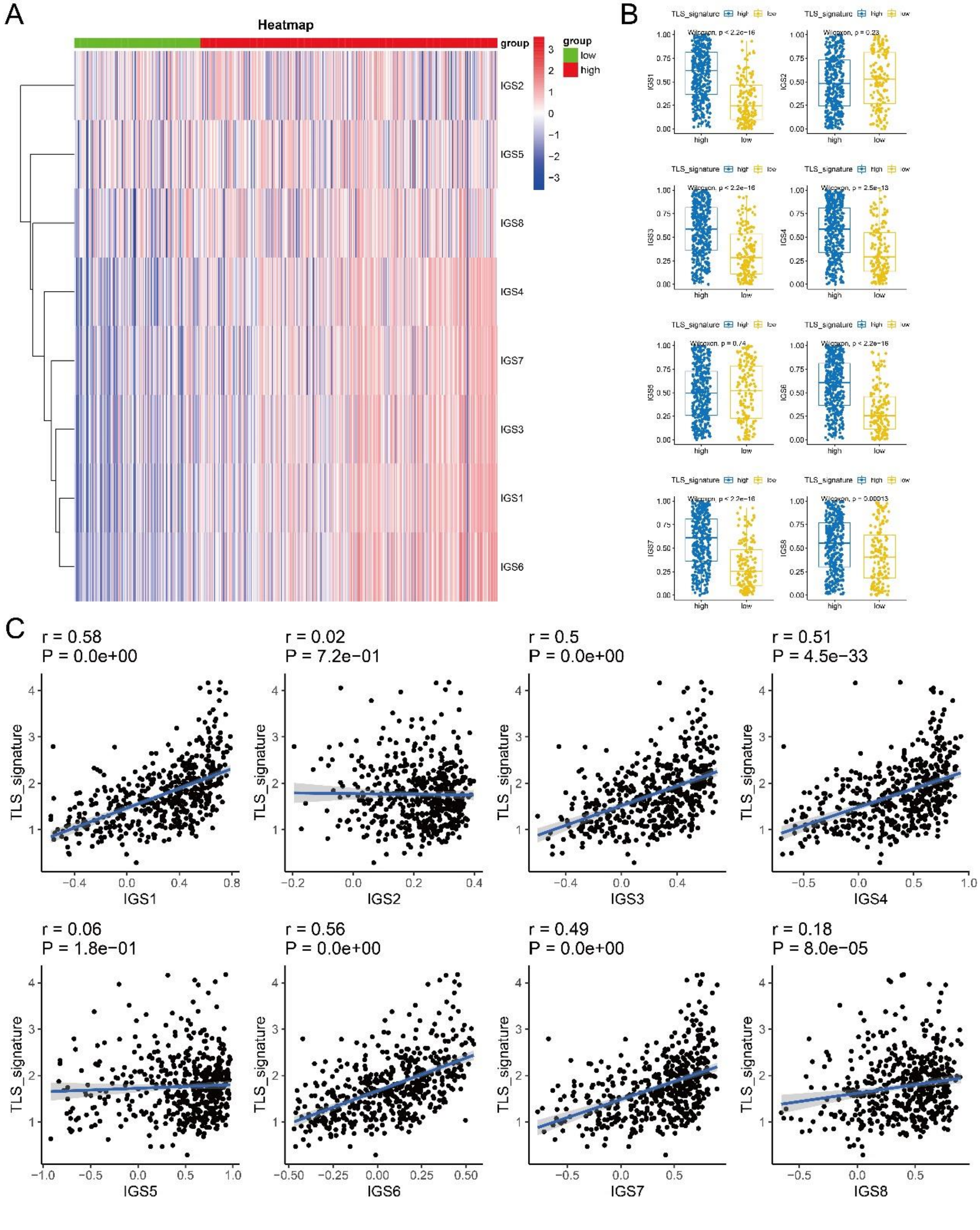

Fig. (S3). Association of TLS signature with immune circulation. A-**B**. Differences in immune circulation between groups with high TLS signature (n = 342) and low TLS signature (n = 146); **C**. Correlation of TLS with various features of immune circulation. P<0.05 was considered a significant difference.

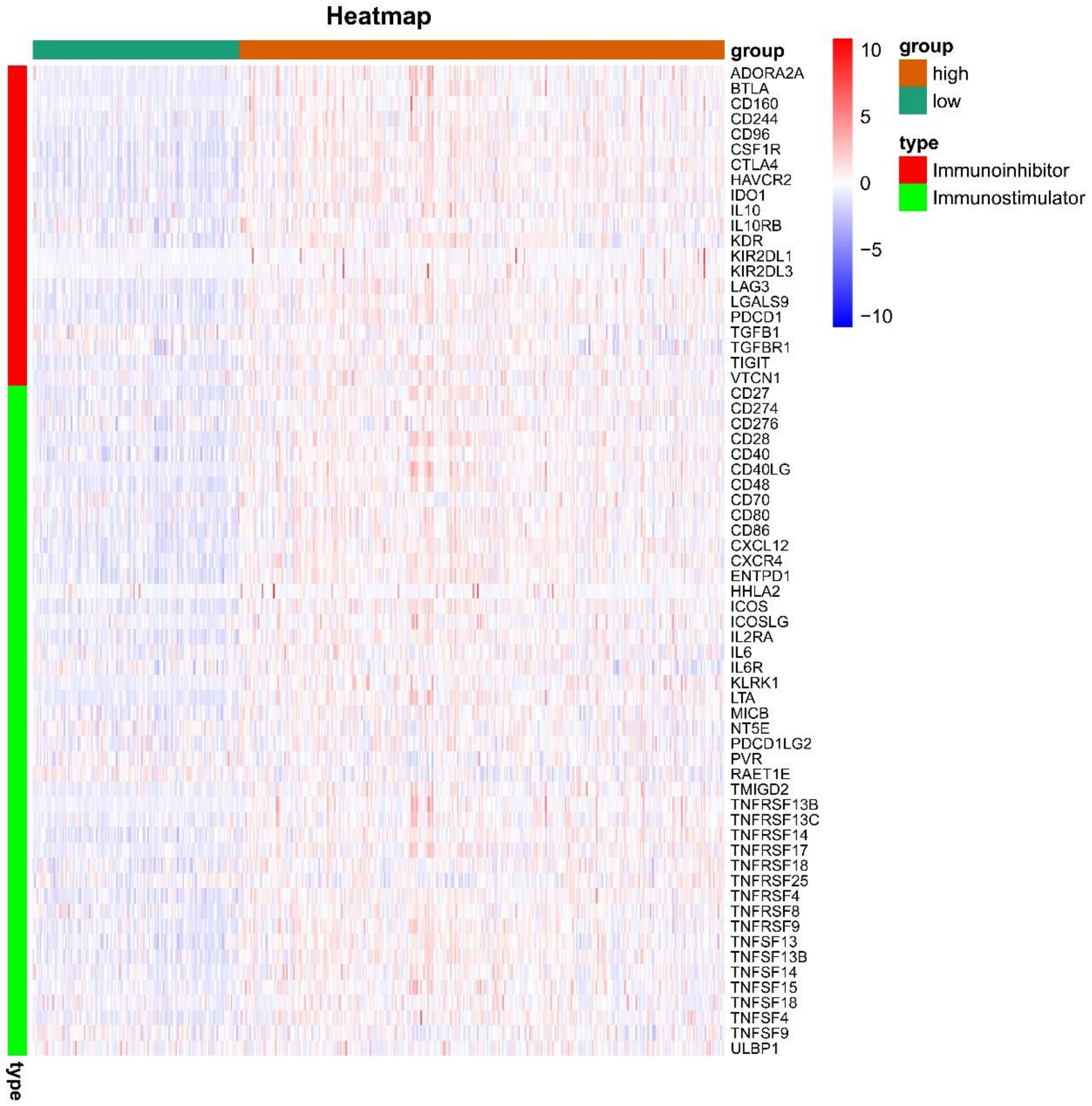

Fig. (S4). Differences in checkpoint gene expression between groups with high/low TLS signature.

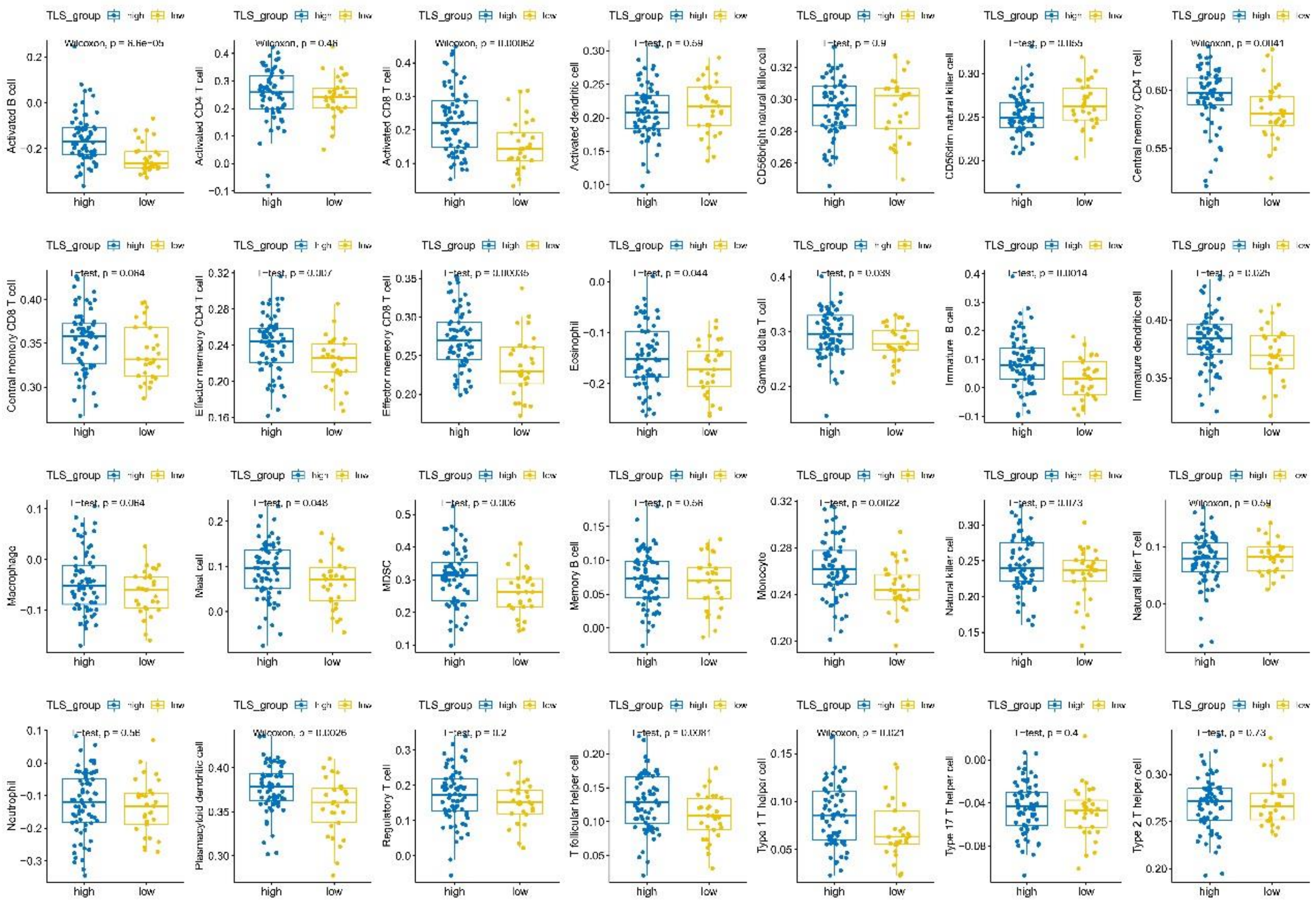

Fig. (S5). Comparison of immune cell subpopulations between groups with high/low TLS signature in GSE41613 dataset.
